# Supplementary material for: Comparative influenza protein interactomes identify the role of plakophilin 2 in virus restriction
Source: Nat Commun. 2017 Feb 7;8:13876. doi: 10.1038/ncomms13876 (PMC5309701; doi:10.1038/ncomms13876)
Supplement: Supplementary Information — Supplementary Figures [file ncomms13876-s1.pdf]

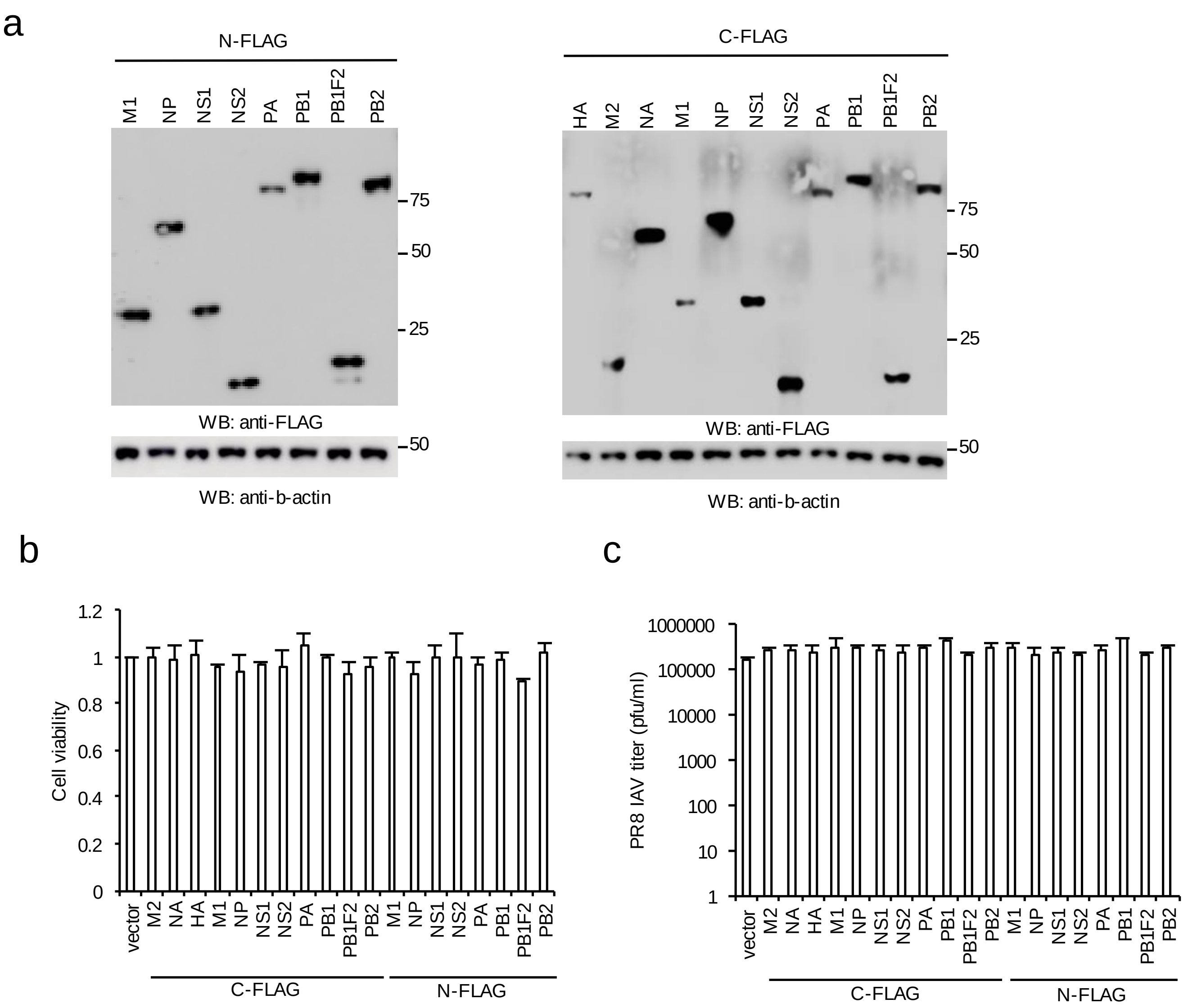

Supplementary Figure 1. Mapping the PR8 IAV-host protein interactome. a, PR8 IAV protein expression levels in the HEK293 stable cell lines. C-FLAG and N-FLAG stand for C-terminal and N-terminal FLAG epitope, respectively. **b**, Cell viability of the HEK293 cell lines stably expressing IAV proteins. CellTiter-Glo Luminescent Cell Viability Assays were performed. The relative cell viability was normalized by the cells transfected with vector control. Data represent means +/− s.d. of three independent experiments. C-FLAG and N-FLAG stand for C-terminal and N-terminal FLAG epitope, respectively. **c**, Viral growth in the indicated stable cell lines. Each stable cell line was infected with 0.001 MOI of PR8 IAV. After 16 hr, virus titers were determined by plaque assay. All experiments were

biologically repeated three times. Data represent means +/− s.d. of three independent experiments. C-FLAG and N-FLAG stand for C-terminal and N-terminal FLAG epitope, respectively.

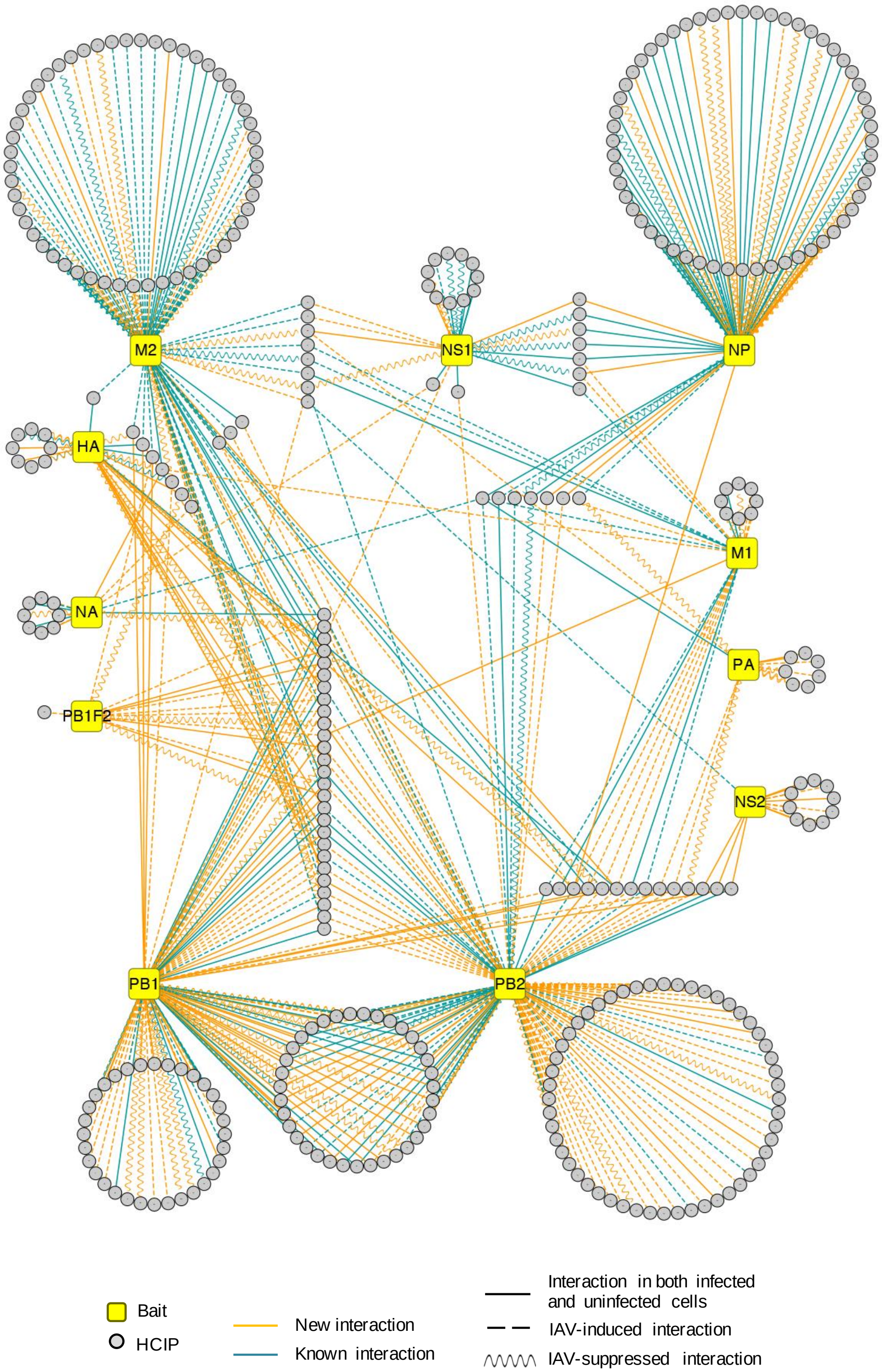

Supplementary Figure 2. Landscape of the PR8 IAV-host protein interactome. Legends are indicated. IAV-induced interaction is the interaction only identified in the samples infected with virus. IAV-suppressed interaction is the interaction only identified in the uninfected samples.

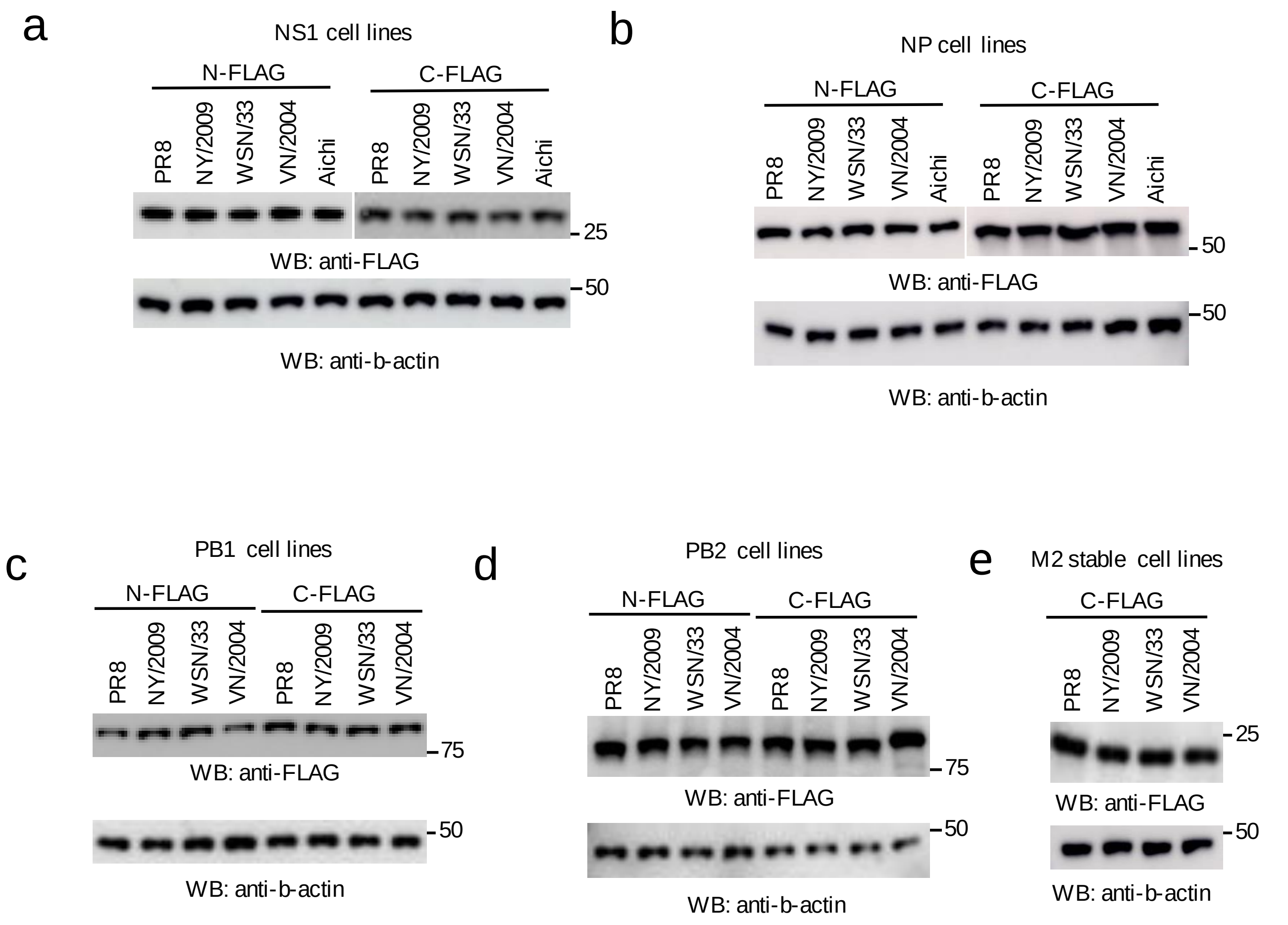

Supplementary Figure 3. IAV protein expression levels in HEK293 stable cell lines. a-e, Western blots determined the protein expression levels of NS1 (**a**), NP (**b**), PB1 (**c**), PB2 (**d**) and M2 (**e**) in HEK293 stable cell lines. C-FLAG and N-FLAG stand for C-terminal and N-terminal FLAG epitope, respectively.

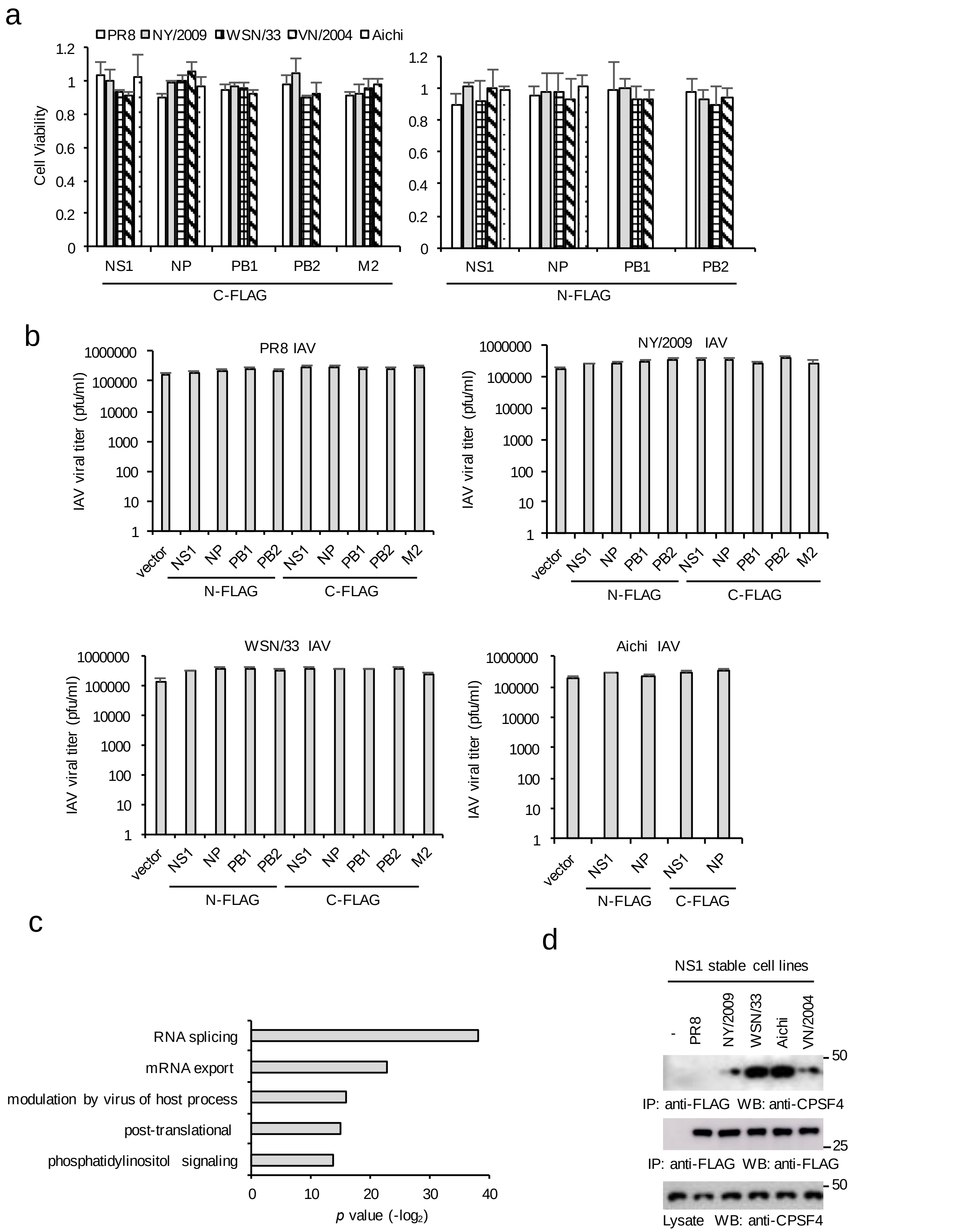

Supplementary Figure 4. Mapping and analysis of IAV-host interactomes. a, Cell viability of the HEK293 cell lines stably expressing IAV proteins. CellTiter-Glo Luminescent Cell Viability Assays were

performed. The relative cell viability was normalized by the cells transfected with vector control. Data represent means +/− s.d. of three independent experiments. C-FLAG and N-FLAG stand for C-terminal and N-terminal FLAG epitope, respectively. **b**, Viral growth in the indicated stable cell lines. Each stable cell line was infected with 0.001 MOI of the corresponding IAV strain. After 16 hr, virus titers were determined by plaque assay. All experiments were biologically repeated three times. Data represent means +/− s.d. of three independent experiments. C-FLAG and N-FLAG stand for C-terminal and N-terminal FLAG epitope, respectively. **c**, Pathways analysis of the 625 HCIPs from IAV-host interactomes by DAVID Bioinformatics Resources 6.8. **d**, The lysates of indicated NS1 stable cell lines were immunoprecipitated with anti-FLAG antibody and blotted as indicated.

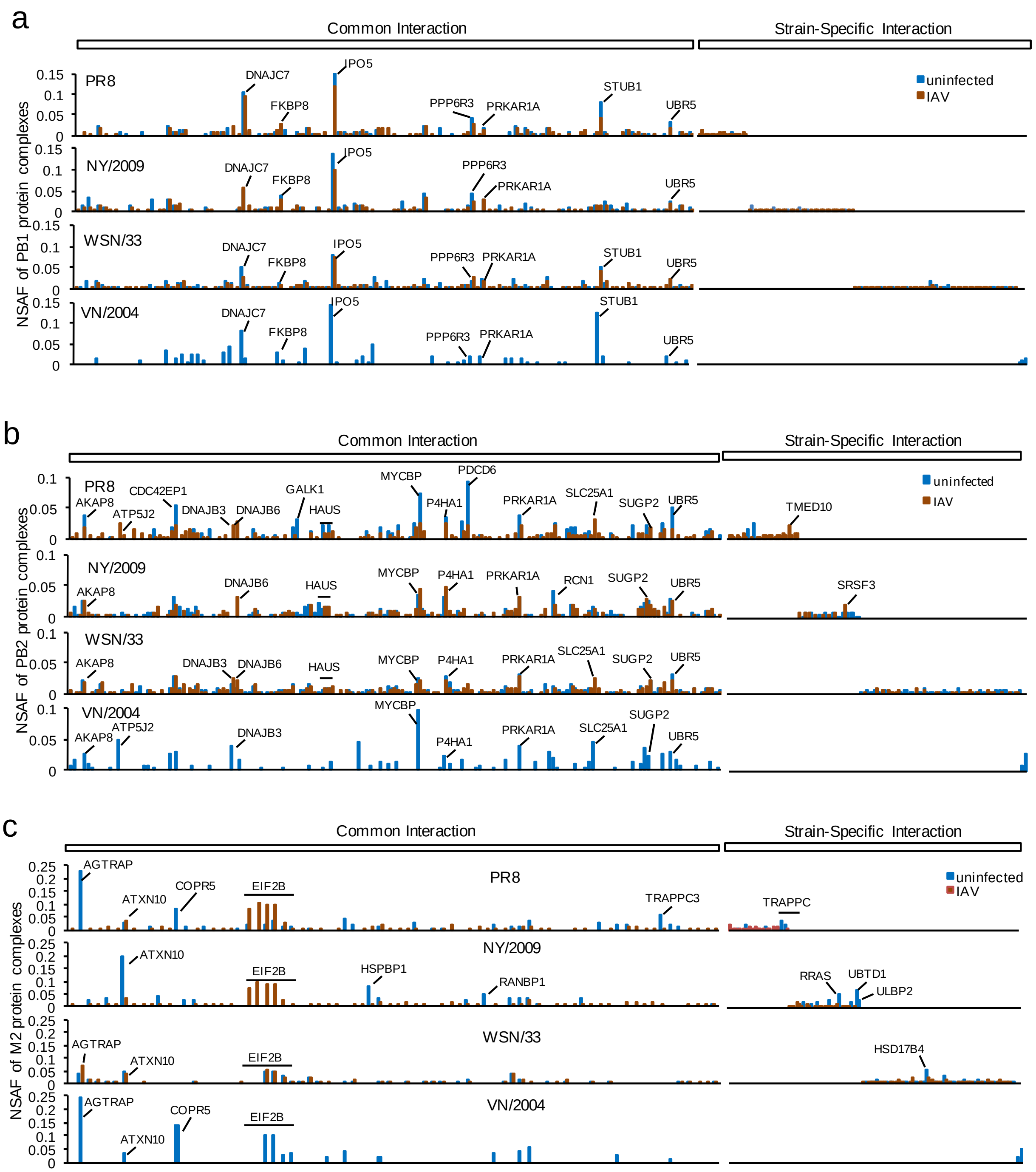

Supplementary Figure 5. Comparative analysis of IAV-host interactomes. a-c, The comparison of relative abundance of HCIPs in each viral protein complex of PB1 (**a**), PB2 (**b**) and M2 (**c**) from the indicated four IAV strains. The outstanding HCIPs are labeled. NSAF stands for normalized spectral abundance factor.

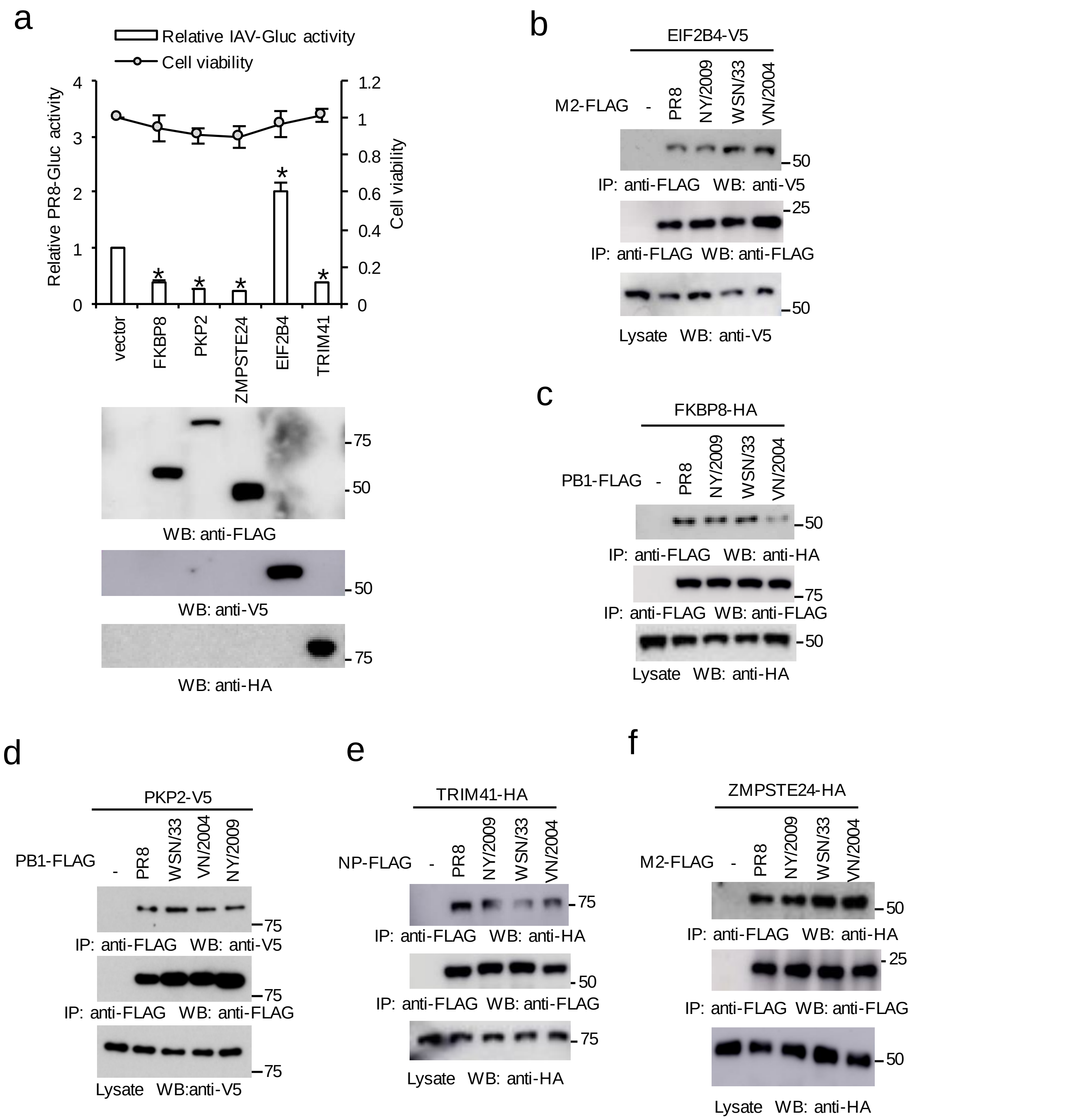

Supplementary Figure 6. Validation of the HCIPs regulating viral infection. a, Validation of the hits from the HCIP functional screening. Indicated candidate genes were transfected into HEK293 cells. After 24 hr, the cells were infected with 0.1 MOI of PR8-Gluc for 16 hr. Luciferase activity assay and CellTiter-Glo Luminescent Cell Viability Assay were performed. The relative viral reporter activity and cell viability were normalized by the vector control. Data represent means +/− s.d. of three independent

experiments. The *P* value was calculated (two-tailed Student's *t*-test) by comparison to the vector control. An asterisk indicates $P<0.05$. Western Blot shows the protein expression levels from one experiment. **b,** EIF2B4-V5 was transfected with the indicated M2-FLAG into HEK293 cells. After 48 hr, cells were harvested and immmunoprecipitated with anti-FLAG antibody, then blotted with indicated antibodies. **c,** FKBP8-HA was transfected with the indicated PB1-FLAG into HEK293 cells. After 48 hr, cells were harvested and immmunoprecipitated with anti-FLAG antibody, then blotted with the indicated antibodies. **d,** PKP2-V5 was transfected with the indicated PB1-FLAG into HEK293 cells. After 48 hr, cells were harvested and immmunoprecipitated with anti-FLAG antibody, then blotted with the indicated antibodies. **e,** TRIM41-HA was transfected with the indicated NP-FLAG into HEK293 cells. After 48 hr, cells were harvested and immmunoprecipitated with anti-FLAG antibody, then probed as indicated. **f,** ZMPSTE24-HA was transfected with the indicated M2-FLAG into HEK293 cells. After 48 hr, cells were harvested and immmunoprecipitated with anti-FLAG antibody, then blotted with the indicated antibodies.

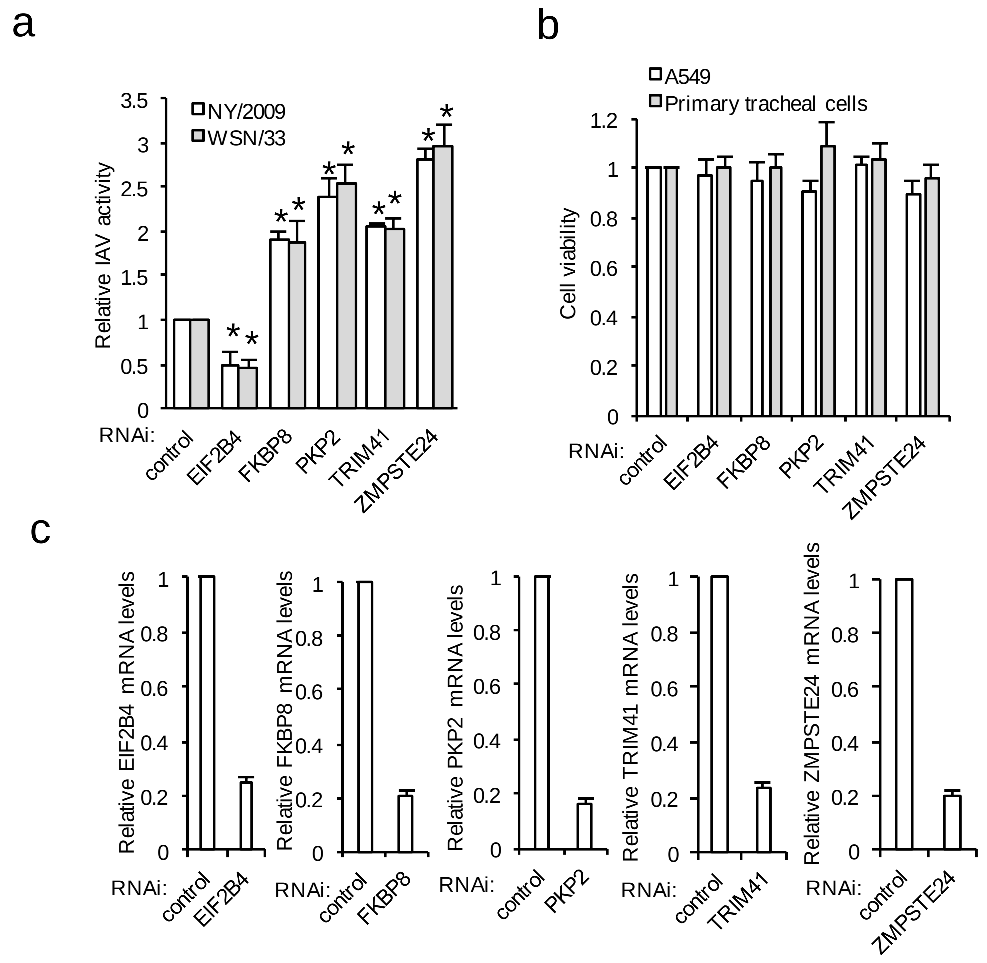

Supplementary Figure 7. RNAi analysis of the effects of HCIPs on IAV infection. a, Primary human tracheal epithelial cells were transfected with control siRNA or the indicated siRNA duplexes. After 48 hr, cells were infected with 1 MOI of WSN/33 or NY/2009 IAV. Fixed cells were stained with anti-NP antibody (WSN/33) or anti-HA (NY/2009) antibody. The relative infection was determined from the ratio of positive cells. Data represent means +/− s.d. of three independent experiments (>80 cells counted per experiment). The *P* value was calculated (two-tailed Student's *t*-test) by comparison to the siRNA control in each cell group. An asterisk indicates *P*<0.05. **b**, The effects of RNAi on cell viability. A549 and primary human tracheal epithelial cells were transfected with control siRNA or the indicated siRNA duplexes. After 72 hr, CellTiter-Glo Luminescent Cell Viability Assays were performed. The relative cell viability was normalized by the RNAi control. Data represent means +/− s.d. of three independent experiments. **c,** RNAi knockdown efficiency. A549 cells were transfected with scrambled

control siRNA or the indicated siRNA oligos. After 72 hr, mRNA levels were determined by real-time PCR. Data represent means +/− s.d. of three independent experiments.

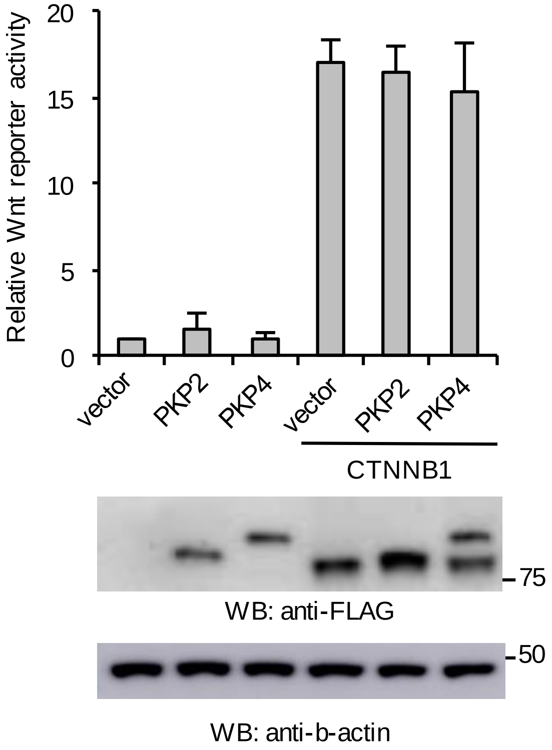

Supplementary Figure 8. The effect of PKP2 on Wnt signaling pathway. HEK293 cells were transfected with pCMV3-tag-8 vector or the indicated genes together with a Wnt pathway reporter, TOPFLASH. After 48hr, cells were lysed and luciferase activities were examined. The relative reporter activity was normalized by the vector control. Data represent means +/− s.d. of three independent experiments.

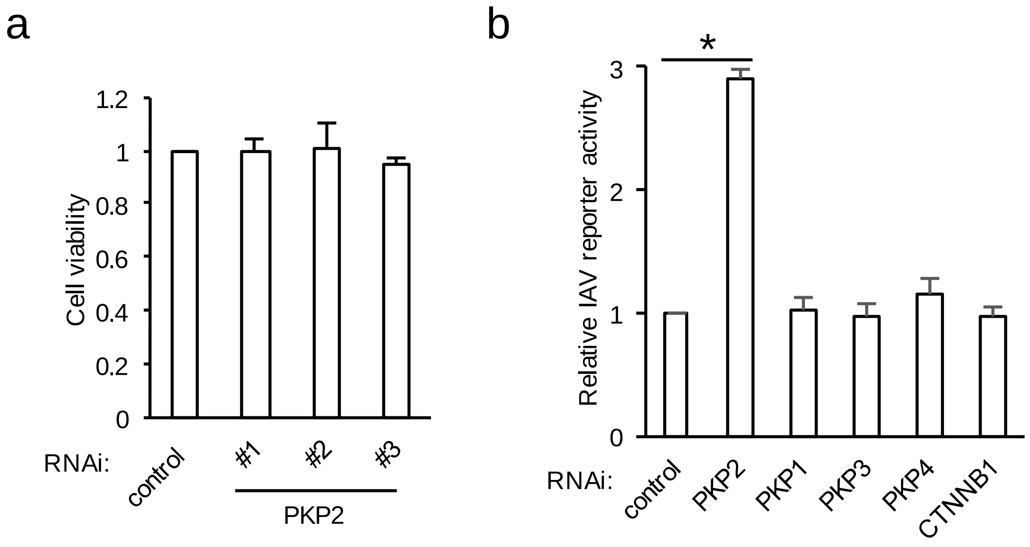

Supplementary Figure 9. Knockdown of PKP2 increases susceptibility to IAV infection. a, The effects of PKP2 siRNA oligos on cell viability. A549 cells were transfected with control siRNA or siRNA oligos against PKP2. After 72 hr, CellTiter-Glo Luminescent Cell Viability Assays were performed. The relative cell viability was normalized by the RNAi control. Data represent means +/− s.d. of three independent experiments. **b,** A549 cells were transfected with scrambled control siRNA or indicated siRNA duplexes. After 48 hr, the cells were infected with 0.1 MOI of IAV PR8-Gluc for 16 hr. Relative luciferase activities were examined. Data represent means +/− s.d. of three independent experiments. The *P* value was calculated (two-tailed Student's *t*-test) by comparison to the siRNA control. An asterisk indicates *P*<0.05.

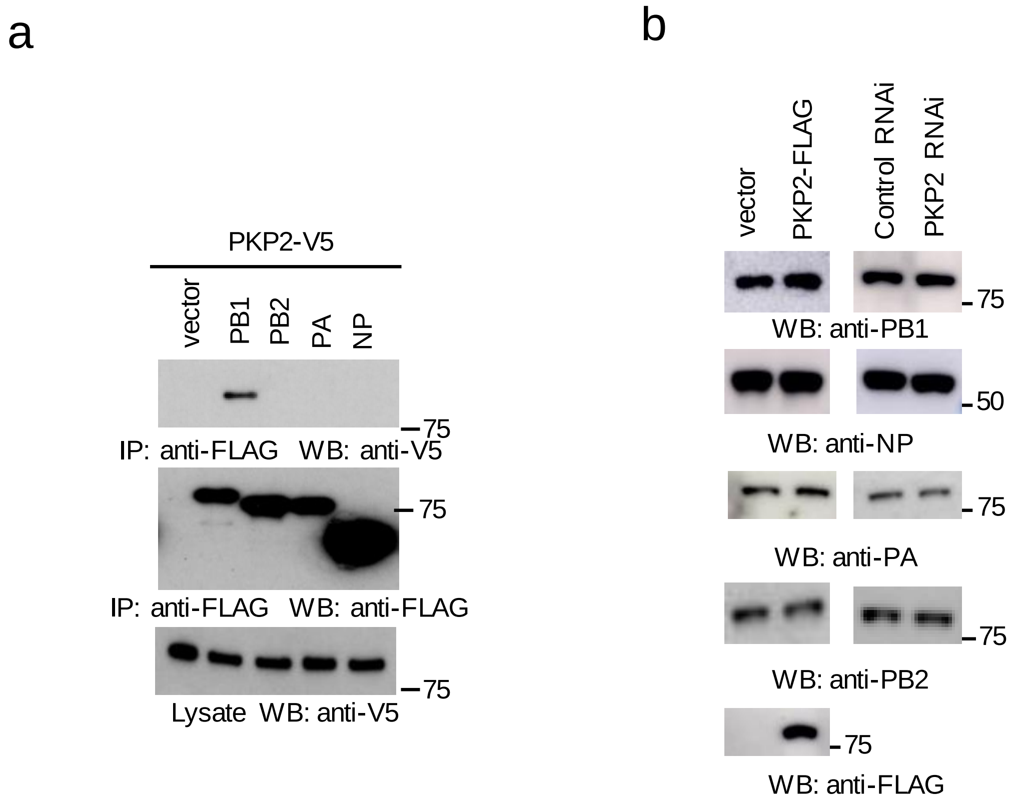

Supplementary Figure 10. PKP2 interacts with PB1 and perturbs IAV polymerase activity. a, PKP2 was transfected into HEK293 cells with NP, PB1, PB2, or PA. After 48 hr, cells were harvested and immunoprecipitated with anti-FLAG antibody, then probed as indicated. **b,** HEK293 cells were transiently transfected with a plasmid cocktail containing PR8 PB1, PB2, PA, NP expression plasmids plus a polymerase I plasmid expressing an influenza virus-like RNA encoding the reporter protein firefly luciferase, along with control siRNA, PKP2 siRNA, the empty vector pCMV3-tag-8 or PKP2-FLAG. After 48 hr, cells were harvested and probed as indicated.

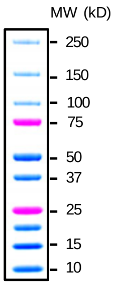

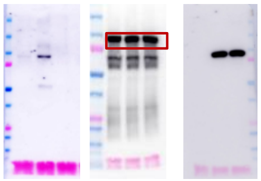

Fig. 3a

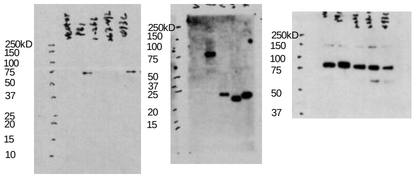

Fig. 3c

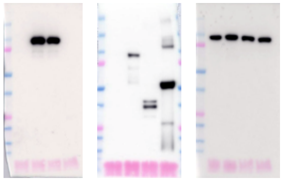

Fig. 3d

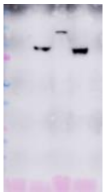

Fig. 4a

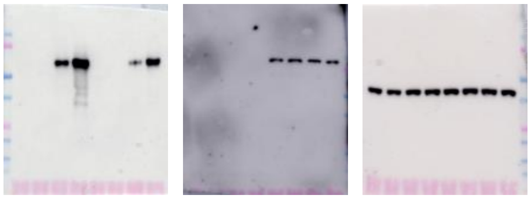

Fig. 4c

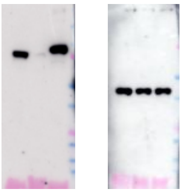

Fig. 5b

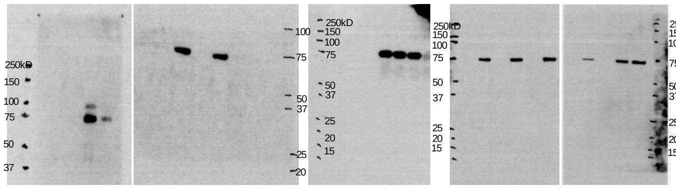

Fig. 6a

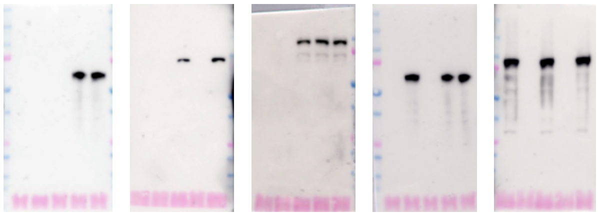

Fig. 6b

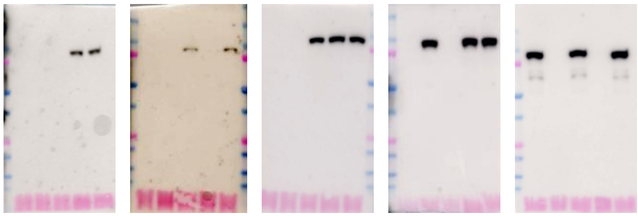

Fig. 6c

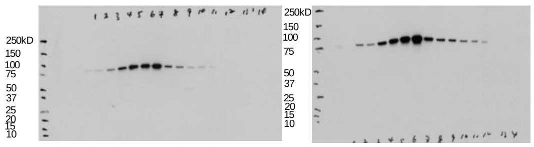

Fig. 6d

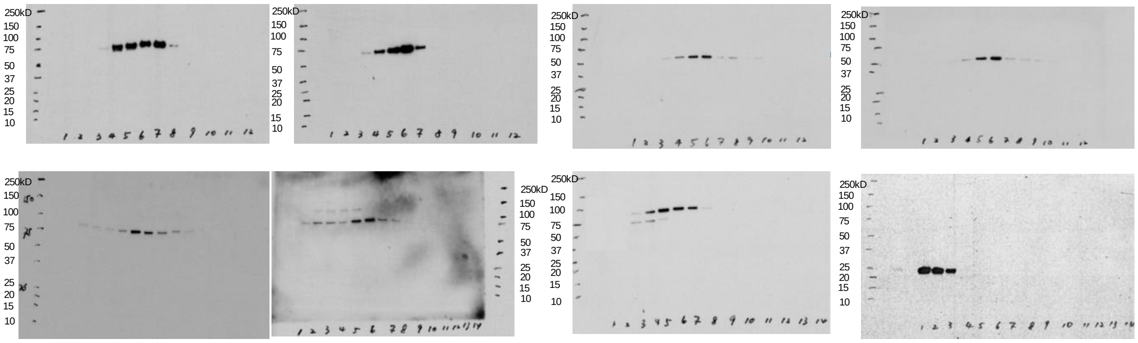

Fig. 6d

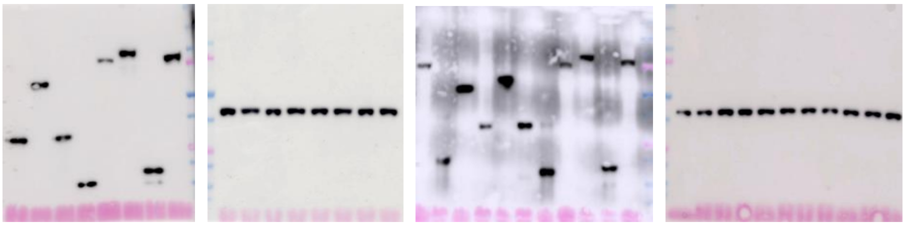

Suppl. Fig. 1b

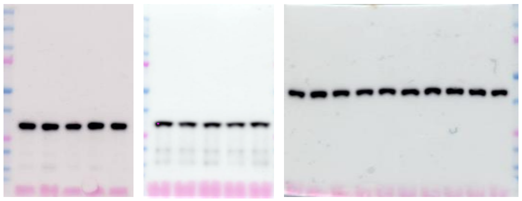

Suppl. Fig. 3a

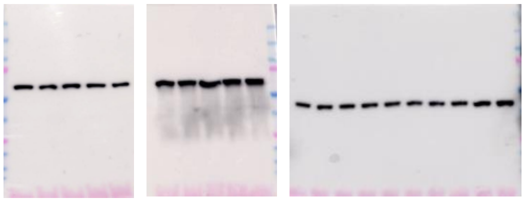

Suppl. Fig. 3b

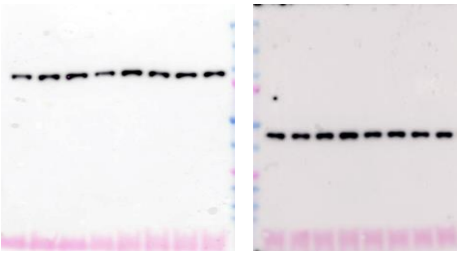

Suppl. Fig. 3c

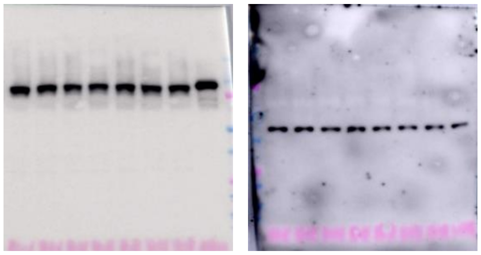

Suppl. Fig. 3d

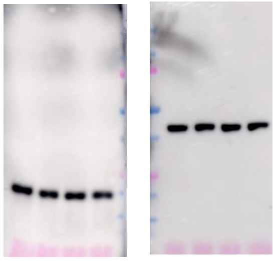

Suppl. Fig. 3e

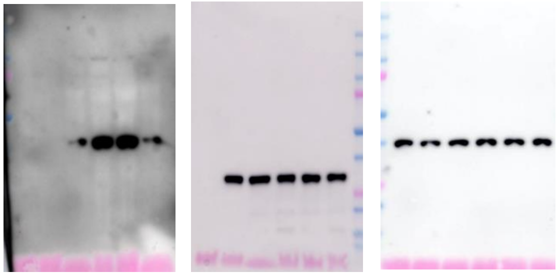

Suppl. Fig. 4d

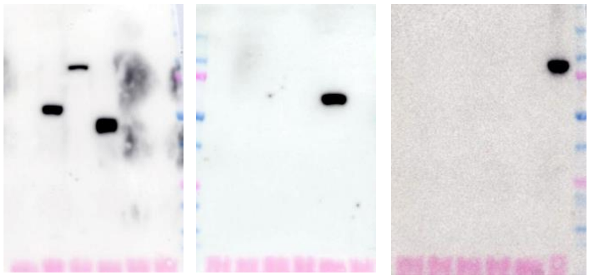

Suppl. Fig. 6a

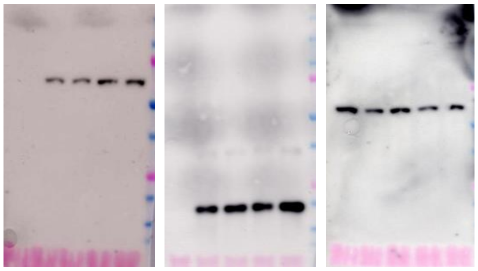

Suppl. Fig. 6b

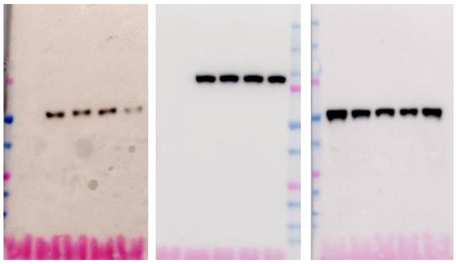

Suppl. Fig. 6c

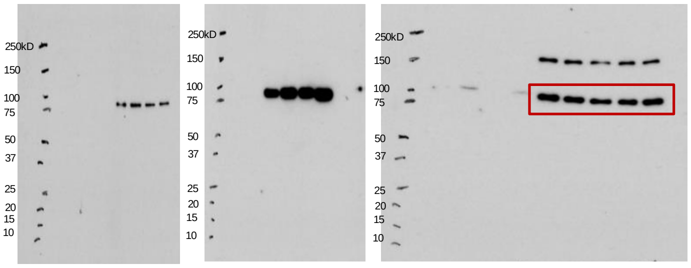

Suppl. Fig. 6d

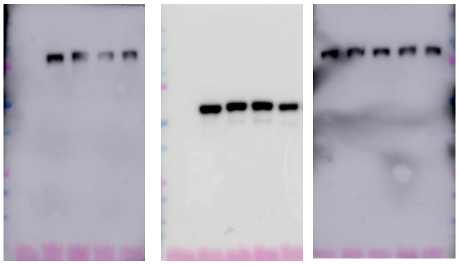

Suppl. Fig. 6e

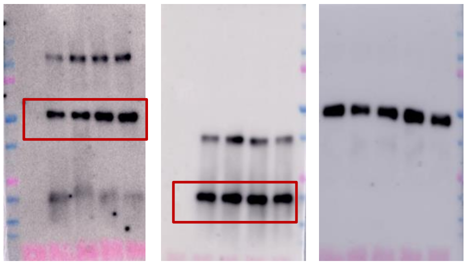

Suppl. Fig. 6f

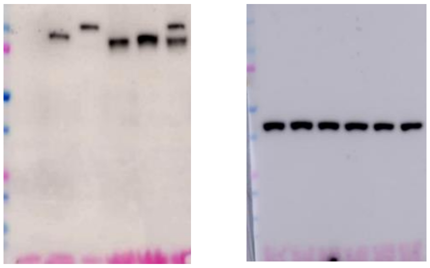

Suppl. Fig. 8

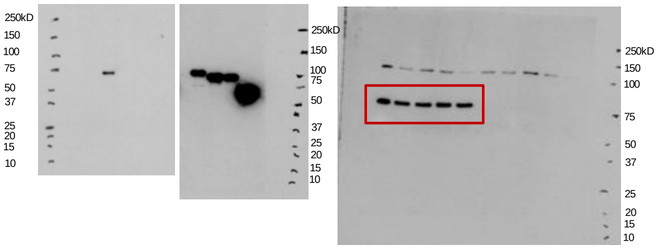

Suppl. Fig. 10a

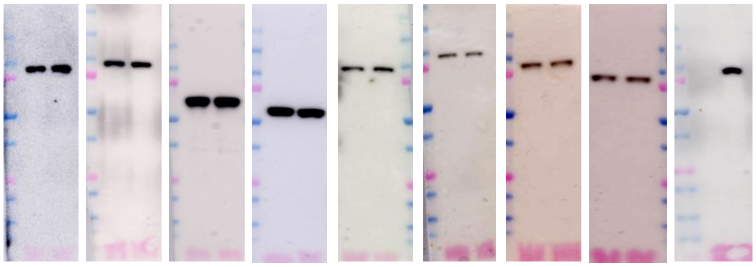

Suppl. Fig. 10b

Supplementary Figure 11. Uncropped immunoblots. Labeling below each panel refers to the corresponding figure in the article. Red frame indicates the bands for which the molecular weight corresponds closest to the molecular weight of the protein of interest.
